# Pattern of acute poisoning at two urban referral hospitals in Lusaka, Zambia

**DOI:** 10.1186/s12873-016-0068-3

**Published:** 2016-01-09

**Authors:** Jessy Z’gambo, Yorum Siulapwa, Charles Michelo

**Affiliations:** Department of Public Health, Epidemiology & Biostatistics Unit, School of Medicine, University of Zambia, Lusaka, Zambia; Department of Public Health, Environmental Health Unit, School of Medicine, University of Zambia, Lusaka, Zambia

**Keywords:** Acute, Poisoning, Chemical, Hospital, Toxic agent, Lusaka, Zambia

## Abstract

**Background:**

Poisoning remains an important public health problem contributing significantly to the global burden of disease. Evidence on the exact burden and pattern of acute poisoning in Zambia is limited. We aimed to characterise acute poisoning with regard to demographic and epidemiologic factors of cases reported at the University Teaching Hospital and Levy Mwanawasa General Hospital; two large referral hospitals in Lusaka, Zambia.

**Methods:**

This was a cross-sectional study involving retrospective collection of data on all poisoning cases recorded in hospital records from 1 January to 31 December 2012. A pretested data collection form was used to extract demographic and other data such as poisonous agents used, circumstance of poisoning, route and outcome of poisoning. All analyses were performed in STATA (StataCorp. 2013. Stata Statistical Software: Release 13. College Station, TX: StataCorp LP).

**Results:**

A total of 873 poisoning cases were reviewed with almost similar proportions of males (52 %) and females (49 %). Poisoning cases were highest in the 0-12 years age category (36 %) followed by the 20-30 years age category (31 %). Accidental poisoning characterised most (65 %) cases in children aged < 13 years. The common route of exposure to poisonous agents was ingestion. Overall, the mortality rate was 2.6 per 100 cases, the majority of deaths were observed in men (78 %). Poisonous agents associated with most cases were pesticides (57 %) and pharmaceuticals (13 %).

**Conclusions:**

The high risk of accidental poisoning observed in children calls for special health education on chemical safety, tailored for mothers and caregivers to prevent chemical exposure in this important age group whose access to toxic agents is mainly in homes or their immediate environment. The results also call for additional regulatory controls on pesticides and pharmaceuticals, which were the most common toxic agents.

## Background

Poisoning remains a significant public health problem associated with over 340 000 unintentional poisoning deaths and an estimated global loss of over 7.4 million years of healthy life (disability adjusted life years, DALYs). Furthermore, there are almost one million suicides each year and a significant number of these deaths are related to chemicals [1, 2]. Low and middle-income countries suffer the highest burden of unintentional and suicidal poisoning – a phenomenon exacerbated by crippled chemicals management structures and health care delivery systems [2]. In Southern Africa, acute poisoning has been identified as a significant cause of both morbidity and mortality with hospital prevalence ranging from 1 to 17 % [3].

As has been reported, more than 25 % of the global burden of disease is linked to environmental factors including exposures to and inappropriate use of toxic chemicals [4]. Current trends show an increase in use of chemicals in the global economy and daily modern life which may be linked to increased human exposure [5, 6]. Limiting the availability of and access to highly toxic chemicals such as pesticides has been shown to reduce the number of deaths due to poisoning. For instance, the withdrawal of all World Health Organisation (WHO) class I pesticides as well as endosulfan through a number of targeted legislative initiatives in Sri Lanka resulted in a 50 % drop in suicides and even greater reduction in fatal poisonings [7, 8]. A call has been made to modify the WHO classification based on new evidence on human lethality from acute poisoning of certain pesticides [7, 8].

Though much is known and documented on acute poisoning globally, the opposite is true for Zambia. The lack of up-to-date information can be attributed to the unavailability of published data in accessible databases, an absence of poison centres and national surveillance systems, including the non-mandatory notification of poisoning cases. Similar challenges have been observed in other countries such as China, Botswana and South Africa [3, 9, 10].

This dearth of information on circumstances, substances and populations at risk is a barrier to effective poisoning prevention and targeted intervention programmes. Therefore, the need for a current review of poisoning patterns in Zambia is imperative.

This study sought to characterise acute poisoning with regard to demographic factors (i.e. age, sex and residence), common toxic agents used and their case fatality rates as well as the overall mortality rate of acute poisoning.

## Methods

### Study area

The study was conducted in Lusaka district, the capital and largest city of Zambia. Lusaka district has a total land area of 375 km^2^ and a total population of 1.7 million inhabitants with almost equal proportions of males and females (i.e. 49 % and 51 % respectively) [11, 12]. The city is Zambia’s most densely populated city with a population density close to 5000 persons per square kilometre. Over 70 % of people in Lusaka district reside in peri urban areas which are characterised by squatter settlements and regularised informal settlements known as Improved Areas [12]. The majority of low income social groups reside in the peri urban areas of Lusaka.

The central location of Lusaka district makes it easily accessible to most parts of the country and provides a ready market for goods and services. The main economic activities in the district are manufacturing, transport, wholesale and retail trading. In addition, despite Lusaka being a built-up urban area and as more productive agricultural land continues to be taken for urban processes, urban agriculture is also an important economic activity consisting of crop cultivation and animal husbandry [12].

Lusaka district is well covered with regard to health care delivery services. The district has 3 third level (i.e. tertiary) hospitals, 1 second level (i.e. secondary) hospital, 9 first level (i.e. primary) hospitals, 170 urban health centres and 11 health post. Of these health facilities, 44 are run by the government while the rest are of private ownership [13]. The University Teaching Hospital (UTH) is a third level referral hospital which caters for a catchment population of approximately over 800 000. Levy Mwanawasa General Hospital (LMGH) is a second level referral hospital intended to cater for a catchment area of between 200 000 and 800 000 people. Both UTH and LMGH receive referral cases from health facilities within Lusaka district as well as from other parts of the country [13]. The first point of contact with the health care service system for the patients presenting with poisoning is at health centres and primary level hospitals. Depending on the level of care required, complicated cases are moved up the chain of care to the secondary (i.e. LMGH) and tertiary hospital (i.e. UTH). However, some patients go straight to the secondary and tertiary level hospitals.

### Population and sampling procedures

The study was cross-sectional and made use of retrospective extraction of data on acute poisoning cases from records at Levy Mwanawasa General Hospital (LMGH) and the University Teaching Hospital (UTH). Filter clinic and the department of paediatrics at UTH provided the adult and children populations respectively. Filter clinic is a medical emergency unit that attends to all medical emergencies in adults including poisoning cases. The department of paediatrics handles all paediatric emergencies including poisoning cases. The department of casualty at LMGH provided both adult and children populations.

### Data collection and extraction

All cases of poisoning recorded in hospital out-patient registers, patient case files and death registers covering a period of one year from 1^st^ January to 31^st^ December 2012 were listed and included in the study. Demographic (i.e. sex, age and residence) and epidemiologic data such as toxic agents used, route of exposure, circumstance (i.e. accidental, deliberate self-harm and recreational) and outcome (i.e. recovery, injury or death) of poisoning were collected using a pretested data collection form. Data collection was done by trained research assistants. The first point of extraction was from out-patient registers which list all patients passing through the selected hospital departments. Serial numbers from out-patient registers were used to locate patient case files containing detailed information about each case. Death registers provided data on all deaths as a result of acute poisoning. Data on route of exposure and circumstance of poisoning were collected as recorded in patient case files. Poisonous agents were described and grouped based on their use, chemical properties and groupings used in other studies.

### Statistical analysis

For quality assurance, data collection forms were checked daily for accuracy, consistency and completeness. Analyses were done in STATA (StataCorp. 2013. Stata Statistical Software: Release 13. College Station, TX: StataCorp LP). Chi Square test was used to examine associations of variables. P ≤ 0.05 was used to determine significance.

## Ethical consideration

Permission was obtained from the hospital administration to access hospital records that contain private information about patients. Patients were only identified by serial numbers as recorded in the hospital registers and all the information was kept confidential. In addition, approval to conduct research was sought from the Excellence in Research Ethics and Science (ERES) Institutional Review Board (IRB) (I.R.B. No. 00005948).

## Results

### Participation and socio-demographic distribution

A total of 1 061 cases were reviewed, 188 of these cases were found to be with incomplete data and were excluded resulting in 873 poisoning cases. The age of patients ranged from 0 to 76 years with a mean age of 22 years (±22 years). Poisoning cases were highest in the 0-12 years age category (36 %) followed by the 20-30 years age category (31 %). Overall, there were almost similar proportions of poisoning cases in males (52 %) and females (49 %) (Table 1). Females had the largest proportion (75 %) of poisoning cases in the 13-19 years age category (Table 2). The majority (64 %) of cases reported were from peri urban areas, others were from urban (27 %) and rural (7 %) residential areas of Lusaka (Table 1). The residence was not known in 1 % of the cases.

**Table 1:** Baseline characteristics of poisoning cases reviewed from all data collection sites, from January to December 2012 (n = 873)

Patient Characteristics	Frequency	Proportion
Sex		
Male	450	51.6
Female	423	48.5
Age		
0 to 12 years	300	36.3
13 to 19 years	149	18.0
20 to 30 years	253	30.6
Over 30 years	125	15.1
Mean age (SD)	22 years (22 years)	
Residence		
Urban	239	27.4
Peri-urban	562	64.4
Rural	60	6.9
Circumstance of poisoning		
Accidental	453	51.9
Deliberate self-harm	336	38.5
Recreational	3	0.3
Outcome of poisoning		
Recovery	847	97.0
Injury	3	0.3
Death	23	2.6
Route of poisoning		
Ingestion	793	90.8
Inhalation	14	1.6
Absorption (Dermal)	1	0.1
Animal/Insect bites	60	6.9
Toxic agent involved		
Household chemicals	44	5.0
Pharmaceutical	123	14.1
Animal/insect venom	60	6.9
Pesticides	187	21.4
Food Poisoning	115	13.2
Narcotics	3	0.3
Traditional medicine	7	0.8
Plants	19	2.2
Unspecified agents	173	19.8
Other agents	142	16.3

• Marital status and occupation were not included in the table because the variables were found to be missing in the hospital records for most cases

• Pharmaceuticals were predominantly oral but difficult to disaggregate

• The circumstance of poisoning for the cases was based on details of information recorded in the case files

• The category ‘other agents’ included chemicals that could not fit into the categories created prior to data collection. The specific agents have been tabulated in Table 5

**Table 2:** Distribution of poisoning cases according to age categories

Patient characteristics	Frequency [Number (%)]			
	0 - 12 years (n = 300 )	13 - 19 years (n = 149 )	20 - 30 years (n = 253 )	> 30 years (n = 125) )
Sex				
Male	177 (59.0)	37 (24.8)	121 (47.8)	84 (67.2)
Female	123 (41.0)	112 (75.2)	132 (52.2)	41 (32.8)
Residence				
Urban	72 (24.7)	46 (30.1)	72 (28.8)	41 (32.8)
Peri urban and rural	220 (75.3)	103 (69.1)	178 (71.2)	84 (67.2)
Route of poisoning				
Ingestion	286 (95.3)	135 (90.6)	226 (89.3)	106 (84.8)
Inhalation	0	4 (2.7)	5 (2)	2 (1.6)
Absorption	1 (0.3)	0	0	0
Animal/insect bites	13 (4.3)	10 (6.7)	19 (7.5)	17 (13.6)
Circumstance of poisoning				
Accidental	288 (98.0)	43 (31.2)	63 (27.8)	49 (49.0)
Deliberate self-harm	6 (2.0)	95 (68.8)	164 (72.3)	51 (51.0)
Outcome of poisoning				
Recovered	300 (100)	148 (99.3)	245 (96.8)	118 (94.4)
Died	0	1 (0.6)	8 (3.16)	7 (5.6)
Toxic agent involved				
Pharmaceutics and narcotics	25 (10.4)	30 (25.0)	51 (24.5)	11 (11.3)
Pesticides	39 (16.3)	32 (26.7)	73 (35.1)	29 (29.9)
Domestic and industrial	98 (40.8)	29 (24.2)	37 (17.8)	15 (15.5)
Plant, animal and food poisoning	78 (32.5)	29 (24.2)	47 (22.6)	42 (43.3)

Category for unknown information not shown for all variables

### Circumstance of poisoning and route of exposure

Most poisoning cases were due to accidental circumstances (52 %, Table 1), only 2 of these cases were linked to occupational chemical exposures. Sixty five percent of accidental poisoning cases were in children aged 0-12 years (Table 3). Deliberate self-harm was associated with 39 % (Table 1) of the poisoning cases and more than half (52 %, Table 3) of these cases were in adults between the ages of 20 and 30 years. Pesticides were the common toxic agents used in deliberate self-harm for most of the cases (43 %, Table 3). Of the cases reviewed, 91 % were exposed to toxins orally while others were exposed by inhalation (2 %), dermal absorption (0.1 %) as well as through animal/insect bites (7 %) (Table 1).

**Table 3:** Factors associated with circumstance of poisoning

Patient characteristics	Circumstance of poisoning [Number (%)]		
	Accidental	DSH^a^	P-Value^*^
Sex			0.002
Male	254 (56.1)	158 (45.1)	
Female	199 (43.9)	186 (54.9)	
Age			< 0.001
0 to 12 years	288 (65.0)	6 (1.9)	
13 to 19 years	43 (9.7)	95 (30.1)	
20 to 30 years	63 (14.2)	164 (51.9)	
Over 30 years	49 (11.1)	51 (16.1)	
Route of poisoning			< 0.001
Ingestion	382 (84.3)	338 (99.7)	
All other routes combined^b^	71 (15.6)	1 (0.21)	
Residence			0.006
Urban	140 (31.4)	76 (22.6)	
Peri urban; rural	306 (68.6)	261 (77.5)	
Outcome of poisoning			< 0.001
Recovered	453 (100)	322 (95.0)	
Died	0	17 (5.0)	
Toxic agent involved			
Pharmaceutics and narcotics	27 (7.0)	93 (33.6)	< 0.001
Pesticides	61 (15.8)	120 (43.3)	
Domestic and industrial	126 (32.7)	55 (19.9)	
Plant, animal and food poisoning	171 (44.4)	9 (3.3)	

^*^P-values were derived using chi square

^a^DSH = Deliberate Self-Harm

^b^Comprised of inhalation, bites/stings and dermal routes

### Acute poisoning mortality

Of all the cases reviewed, 23 had died representing a mortality rate of 2.6 per 100 cases. There were 3 injuries recorded and these were predominantly oesophageal injuries which were a result of damage caused by corrosive chemicals ingested by the patients. Death in men was as high as 78 % while only 22 % of the deaths were observed in females (p = 0.009) (Table 4). There were no deaths observed in children during the period reviewed and none of those who were accidentally poisoned had died. Most of the deaths were observed in patients of the age categories 20-30 years (50 %) and over 30 years (44 %) (p < 0.001, Table 4). The most common route of exposure to toxic chemicals for patients who had died was ingestion (74 %). The majority (73 %) of those who died resided in peri urban and rural areas of Lusaka (Table 4).

**Table 4:** Factors associated with outcome of poisoning

Patient characteristics	Outcome of poisoning [Number (%)]		
	Recovery	Death	P-Value^*^
Sex			0.009
Male	432 (50.8)	18 (78.3)	
Female	418 (49.2)	5 (21.7)	
Age			< 0.001
0 to 12 years	300 (37.0)	0	
13 to 19 years	148 (18.3)	1 (6.25)	
20 to 30 years	245 (30.2)	8 (50.0)	
Over 30 years	118 (14.6)	7 (43.8)	
Route of poisoning			0.004
Ingestion	776 (91.3)	17 (73.9)	
All other routes combined^a^	74 (8.7)	6 (26.1)	
Residence			0.959
Urban	233 (27.8)	6 (27.3)	
Peri urban; Rural	606 (73.2)	16 (72.7)	
Circumstance of poisoning			< 0.001
Accidental	453 (58.5)	0	
Deliberate self-harm	322 (41.6)	17 (100)	
Toxic agent involved			< 0.001
Pharmaceutics and narcotics	122 (17.9)	4 (22.2)	
Pesticides	174 (25.5)	13 (72.2)	
Domestic and industrial	185 (27.1)	1 (5.6)	
Plant, animal and food poisoning	201 (29.5)	0	

^*^P - values were derived using chi square

^a^Comprised of inhalation, bites/stings and dermal routes

### Toxic agents and their case fatality rates

**Fig. 1:**
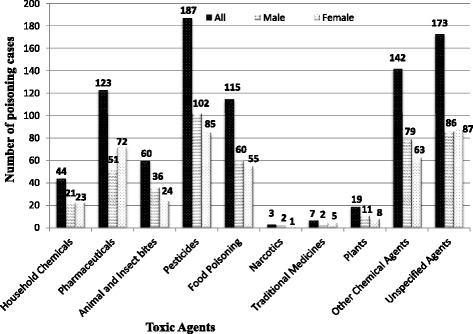
Distribution of toxic agents by sex

Overall, pesticides (57 %) and pharmaceuticals (13 %) were associated with a larger proportion of deaths, representing case fatalities of 7 % and 2 % respectively. Narcotics were responsible for 4 % of the poisoning cases with a lone fatality (33 % case fatality). Poisoning by pesticides was more prevalent in men (n = 102, 55 %) compared to females (n = 85, 45 %) (Fig. 1). On the contrary, poisoning by pharmaceuticals was more prevalent in females (59 %) particularly among young adults (51 %) and teenagers (34 %) (Table 2). Of the industrial chemicals identified, kerosene (49 %) was more predominant and the majority of cases (86 %) were in children in the age category of 0-12 years (results not shown). The toxic agent involved in poisoning could not be identified in a significant proportion of cases (20 %). The primary toxicant of interest was recorded in 25 cases were the patients ingested more than one agent. In 17 of these cases, alcohol (unspecified) was ingested with pesticides (n = 13) and pharmaceuticals (n = 4). All the toxic agents involved (with an exception of alcohol) in such cases were listed and included in Table 5 which outlines all the specific toxic agents involved in poisoning.

**Table 5:** Specific description of agents involved in poisoning cases reviewed

Toxic agent	Frequency n (%)
Narcotic drugs (n = 3)	
Amphetamines	2 (66.7)
Glue^a^	1 (33.3)
Plants (n = 19)	
Unspecified plants	14 (93.7)
Elephant ear plant^b^	5 (26.3)
Household chemicals (n = 44)	
Disinfectants	19 (43.2)
Cleaning agents	15 (34.1)
Personal care products	8 (18.2)
Food additives	2 (4.6)
Animal/Insect bites (n = 60)	
Snake	53 (88.3)
Bee	4 (6.7)
Wasp	2 (3.3)
Scorpion	1 (1.7)
Pharmaceuticals (n = 128^c^)	
Analgesics	56 (43.8)
Antibiotics	20 (15.6)
Unspecified drugs	18 (14.6)
Antipsychotics	7 (5.5)
Nutrition supplements	6 (4.7)
Anticonvulsants	6 (4.7)
Antimalarial drugs	6 (4.7)
Anti-retroviral drugs	4 (3.1)
Antihistamines	2 (1.6)
Family planning pills	1 (0.8)
Antihypertensive drugs	1 (0.8)
Anti-Tuberculosis (TB) drugs	1 (0.8)
Pesticides (n = 187)	
Unspecified organophosphates	97 (51.9)
Insecticides	76 (40.6)
Rodenticides	14 (7.5)
Other agents (n = 142)	
Kerosene	69 (48.6)
Carbon monoxide	26 (18.3)
Acids	23 (16.2)
Construction chemicals	11 (7.8)
Spirit of salt	6 (4.3)
Formalin	3 (2.1)
Diesel	1 (0.7)
Car radiator cooler	1 (0.7)
Brake fluid	1 (0.7)
Silica gel	1 (0.7)

^a^A volatile solvent/inhalant

^b^A plant of the genus *Colocasia*

^c^Counts for both single and combination drug overdose cases

## Discussion

In the present study, it was observed that the majority of poisoning cases were in children involving accidental circumstances. Literature shows that although accidental poisoning can occur at any age, it is most common in children with peak age around two years [10, 14–16]. Hand to mouth behaviour of inquisitive children as they explore the world around them, coupled with the lack of knowledge of consequences puts the children at a higher risk of poisoning [2, 17]. Overall, non sex differentiation was observed in the distribution of poisoning cases reviewed in this study. Age and sex distribution of poisoning burden vary in different geographic regions and time periods due to the interaction and influence of socioeconomic, cultural and behavioural factors in the general population [17–19].

The mortality rate of 2.6 per 100 cases noted in this study was similar to findings in a study conducted in South Africa [3]. A male predominance in deaths was observed in the poisoning cases reviewed in this study. This pattern has been observed by others and has been attributed to the male tendency to choose more violent and successful means of self-harm than women [16, 19]. Furthermore, high case fatality rates were associated with pesticide poisoning. This observation can be attributed to the high toxicity of these agents. However, other toxicological factors - such as potency of toxic agent and amount exposed to - also need to be put into consideration with regard to survival of the victims. While mortality is usually high in patients of deliberate self-harm, a study in rural Sri Lanka found that the choice of poison was based on availability and not toxicity of the poison [20].

The finding that the most prevalent chemical agents involved in poisoning were pesticides and pharmaceuticals was not surprising because these tend to be the most predominant chemicals in poor resource settings. Existing literature shows that toxic agents associated with morbidity and mortality are influenced by various factors such as location, time periods, availability and use of chemicals or poisoning agents, as well as changes in lifestyles, beliefs and traditions of people [21, 22]. To this effect, we observed similarities and differences in findings from our study and those found by others. For instance, a Zimbabwean study revealed that pesticides and pharmaceuticals were the most common toxic agents responsible for hospital admissions [16]. In Francistown and Gaborone, Botswana, household chemicals and pharmaceuticals were the predominant cause of acute poisoning [3]. In Kampala, Uganda, agrochemicals, household chemicals and carbon monoxide were more prevalent among the cases [23]. A study conducted in Hong Kong found sleeping pills and analgesics to be the most common poisons [9]. In Khuzestan region, South Western Iran, envenomation by scorpions, spiders and snakes was the major cause of poisoning [24].

Though only a few cases with narcotic poisoning were recorded in the present study, this observation is of particular importance since Zambia has in recent years become a consumer of hard drugs as evidenced by the increasing number of drug-dependent persons attended to by the Drug Enforcement Commission [25]. This increase in the rate of drug abuse observed poses a threat to public health in the near future. In addition, our study showed that children recorded more cases of poisoning with kerosene. This finding is in support of evidence in the literature that kerosene and paraffin oils are often kept in unsafe non-child-proof containers, resulting in accidental ingestion of the chemicals by children [17].

The number of snake envenomation cases recorded in this study was in accordance with those recorded in studies within countries such as Zimbabwe, Uganda and South Africa where more than 10 % were affected [16, 23]. As noted by the WHO, snake bites are an important public health issue in rural areas of sub-Saharan African countries - like Zambia - where the burden of snakebites is also high [26].

Background factors such as residence and socioeconomic status have been associated with acute poisoning elsewhere [27, 28]. Residential areas in urban Lusaka are classified as high, medium and low-cost housing areas based on the infrastructure and social services available. Studies and surveys have shown distinct differences in socioeconomic characteristics among these classes such as level of education, income and employment status [29]. A gradient in number of cases was observed with regard to area of residence. Most of the cases were from low cost, densely populated residential areas suggesting an influence of socioeconomic status and living conditions on acute poisoning.

## Limitations

The use of secondary data in the study limited control over the quality of data that were collected due to unsatisfactory record-taking and record-keeping. Pages from registers were torn out and some patient files were missing. In the files that were available, certain information such as marital status and occupation was not entered, though provision was made for the collection of such data in the registers. A good number of toxic agents were not specified in the records, making their classification difficult. Also, for most toxic agents identified, only the generic name was indicated which made the classification difficult. Furthermore, this being a hospital-based study we acknowledge that the results may not be representative of the general population. Data from the coroner’s reports was not included in the study which may result in omission of some of the deaths which may have been due to poisoning.

Considering that the likelihood of being referred to higher level hospitals increases with clinical complexity and severity of poisoning cases, we acknowledge that some cases could have been missed as not all cases were referred to the study hospitals. This has further implications in that, deaths could be over represented in the study hospitals because they are more likely to receive severe cases. Also, due to their sensitive and delicate nature, children stand a higher chance of being referred as compared to adults and could therefore be over represented in the study hospitals. However, this does not undermine the findings in this study as referral hospitals have been shown to be good surrogates for monitoring poisoning in a wider population base [30]. Hence, the information provided is still valuable in describing the pattern of acute chemical poisoning in Lusaka.

## Conclusions

It can be concluded that children, whose circumstance of poisoning is mainly unintentional, are at a high risk of poisoning. The study also revealed that although deliberate self-harm was common in young adult females, mortality was higher in males. Most poisoning cases were from the social demographically disadvantaged peri urban areas of Lusaka district. The findings of this study identify the need for health education in the general public on chemical safety, particularly with regard to pesticides and pharmaceuticals to prevent morbidity and mortality due to this problem. Special health education on chemical safety may be tailored for mothers and caregivers to prevent chemical exposure in children whose access to toxic agents is known to be mainly in homes or their immediate environment. In addition, information on the prevention of snake bites may be incorporated in public health messages, especially during the rainy season when most snake bites generally occur. Health education may be supported by strengthening and enforcing regulations addressing the control of pharmaceuticals, pesticides and other chemicals to prevent unnecessary access and exposure.

Further prospective studies are required to explore the pattern of poisoning in other geographical locations of the country and for longer time periods. This may aid in creation of models for predicting poisoning in the different regions which may provide for effective diagnosis, management and prevention of poisoning in Zambia.

## Abbreviations

DALY’s: Disability Adjusted Life Years
ERES: Excellence in Research Ethics and Science
IRB: Institutional Review Board
LMGH: Levy Mwanawasa General Hospital
UTH: University Teaching Hospital
WHO: World Health Organisation

## References

[1] World Health Organization WHO (2008). Global Burden of Disease: 2004 update.

[2] World Health Organisation WHO. Poisons information, prevention and management. http://www.who.int/ipcs/poisons/en/ (2014). Accessed 05 Jun 2014.

[3] Malangu N (2008). Characteristics of acute poisoning at two referral hospitals in Francistown and Gaborone. SA Fam Pract.

[4] World Health Organisation WHO (2009). Manual for the Public Health Management of Chemical Incidents.

[5] Chemicals Abstract Services CAS. Content at a Glance. http://www.cas.org/content/at-a-glance (2014). Accessed 16 Jun 2014.

[6] Organisation for Economic Co-operation and Development OECD. Environmental Outlook for the Chemicals Industry. http://www.oecd.org/dataoecd/7/45/2375538.pdf (2001). Accessed 15 Jun 2014.

[7] Dawson AH, Eddleston M, Senarathna L, Mohamed F, Gawarammana I, Bowe SJ (2010). Acute human lethal toxicity of agricultural pesticides: a prospective cohort study. Plos Med.

[8] Miller M, Bhalla K (2010). An urgent need to restrict access to pesticides based on human lethality. Plos Med.

[9] Chan YC, Fung HT, Lee CK, Tsui SH, Ngan HK, Sy MY (2005). A prospective epidemiological study of acute poisoning in Hong Kong. Hong Kong J emerg med..

[10] Veale DJ, Wium CA, Muller GJ (2013). Toxicovigilance. I: A survey of acute poisonings in South Africa based on Tygerberg Poison Information Centre data. S Afr Med J.

[11] Central Statistics Office (CSO). Zambia 2010 Census of Population and Housing: Preliminary Population Figures. 2011. http://unstats.un.org/unsd/demographic/sources/census/2010_PHC/Zambia/PreliminaryReport.pdf. Accessed 15 Jun 2014.

[12] United Nations Human Settlements Programme (UN-HABITAT). Zambia: Lusaka Urban Profile. Nairobi: UNON, Publishing Services Section; 2007.

[13] Republic of Zambia. The 2012 List of Health Facilities in Zambia: Preliminary Report (Version No. 15). http://www.moh.gov.zm/docs/facilities.pdf (2013). Accessed 20 October 2015.

[14] Jepsen F, Ryan M (2005). Poisoning in Children. Current Paediatrics..

[15] Malangu N, Ogunbanjo GA (2009). A profile of acute poisoning at selected hospitals in South Africa. South African Journal of Epidemiology Infection.

[16] Tagwireyi D, Ball DE, Nhachi CF (2002). Poisoning in Zimbabwe: a survey of eight major referral hospitals. J Appl Toxicol.

[17] Eddleston M (2000). Pattern and problems of deliberate self-poisoning in the developing world. QJ Med..

[18] Hawton K, Fagg J (1992). Trends in deliberate self poisoning and self injury in Oxford, 1976-90. BMJ.

[19] Camidge DR, Wood RJ, Bateman DN (2003). The epidemiology of self-poisoning in the UK. British Journal of Clinical Pharmacology.

[20] Eddleston M, Karunaratne A, Weerakoon M, Kumarasinghe S, Rajapakshe M, Sheriff MH (2006). Choice of poison for intentional self-poisoning in rural Sri Lanka. Clin Toxicol (Phila).

[21] Joint WHO/IPCS/CEC Meeting, Kulling P., La Ferla F. Prevention of Acute Chemical Poisoning: High-Risk Circumstances: Munster, 8-12 December 1986. Copenhagen, Europe ROf;1986.

[22] Thundiyil JG, Stober J, Besbelli N, Pronczuk J (2008). Acute pesticide poisoning: a proposed classification tool. Bull World Health Organ.

[23] Malangu N (2008). Acute poisoning at two hospitals in Kampala-Uganda. Journal of Forensic and Legal Medicine..

[24] Jalali A, Savari M, Dehdardargahi S, Azarpanah A (2012). The pattern of poisoning in southwestern region of Iran: envenoming as the major cause. Jundishapur J Nat Pharm Prod.

[25] Drug Enforcement Commission (DEC). Drug Enforcement Commission: Annual Report (2011-2012). http://www.deczambia.gov.zm/downloads/2011-2012%20ANNUAL%20REPORT.pdf (2014). Accessed 28 Jun 2014.

[26] World Health Organisation WHO. Snake Antivenoms. www.who.int/mediacentre/factsheets/fs337/en/ (2010). Accessed 05 Jun 2014.

[27] Chang SS, Sterne JA, Wheeler BW, Lu TH, Lin JJ, Gunnell D (2011). Geography of suicide in Taiwan: spatial patterning and socioeconomic correlates. Health Place.

[28] Harriss L, Hawton K (2011). Deliberate self-harm in rural and urban regions: a comparative study of prevalence and patient characteristics. Soc Sci Med.

[29] Mweembaa MJA, Webb E (2008). Residential area as proxy for socio-economic status, paediatric mortality and birth weight in Lusaka.

[30] Senarathna L, Buckley NA, Jayamanna SF, Kelly PJ, Dibley MJ, Dawson AH (2012). Validity of referral hospitals for the toxicovigilance of acute poisoning in Sri Lanka. Bull World Health Organ.

